# Effect of Maladaptive Beliefs and Attitudes about Sleep among Community-dwelling African American Men at Risk for Obstructive Sleep Apnea

**DOI:** 10.4172/2167-0277.1000269

**Published:** 2017-05-20

**Authors:** Natasha J Williams, Girardin Jean-Louis, Mirnova E Ceïde, Abishek Pandey, Ricardo Osorio, Mary Mittelman, Samy I McFarlane

**Affiliations:** 1 Center for Healthful Behavior Change, Division of Health and Behavior, Department of Population Health NYU School of Medicine, New York, USA; 2 Montefiore Medical Center, Department of Psychiatry and Behavioral Science, Bronx, New York; 3 Department of Medicine, State University of New York-Downstate Medical Center, Brooklyn, New York, USA; 4 Department of Psychiatry NYU School of Medicine, New York, New York, USA; 5 Department of Psychiatry and Department of Rehailitation Medicine, NYU School of Medicine, New York, USA; 6 Department of Medicine, Division of Endocrinology State University of New York-Downstate Medical Center, Brooklyn, New York, USA

**Keywords:** Beliefs about sleep, Obstructive sleep apnea, Black, African american, Race, Men, Insufficient sleep

## Abstract

This study compared differences in both maladaptive beliefs and attitudes about sleep between African American (heareafter referred to as black) men at risk for obstructive sleep apnea (OSA) and those without OSA risk.

**Methods:**

A convenience sample of 120 community-dwelling men provided sociodemographic, health and sleep data. A validated questionnaire was used to identify men at high risk for OSA and the Dysfunctional Beliefs and Attitudes about Sleep (DBAS-16) scale was used to measure endorsed attitudes and beliefs about sleep.

**Results:**

The mean age of the sample was 42 ± 15 years. Men reported difficulty falling asleep (23%), difficulty maintaining sleep (23%), early morning awakening (35%), and use of sleep medicine (6%). 27% were at high risk for OSA. Men at high OSA risk had greater DBAS scores [F1, 92=13.68, p<0.001]; OSA risk was related to greater rate of sleep dissatisfaction overall [46% *vs*. 13%, Χ2=24.52, p<0.001].

**Conclusion:**

The findings suggest that maladaptive beliefs and attitudes about sleep are important characteristics of black men at risk for OSA, and potential screenings around sleep difficulties should also consider these factors.

## Introduction

Obstructive sleep apnea (OSA) is a debilitating chronic condition characterized by snoring and repetitive partial or complete obstruction of the upper airway. It is estimated to affect approximately 18 million adults in the US. Risk factors for OSA include habitual snoring, older age, craniofacial abnormalities, alcohol consumption, and obesity [1]. Men are at greater risk for OSA and the ratio of OSA in men to women in the general population is estimated to be 5 to 1 [2].

Black race is also reported to be a risk factor for OSA [3,4] with reports of higher prevalence of OSA among Blacks. However, this finding is not consistent across all age groups. Moreover, there is some evidence to suggest that blacks are underdiagnosed [5] and because of the limited population level data 1, the prevalence of OSA in this population is poorly characterized.

A few studies have also examined the severity of OSA by race/ethnicity. Pranathiageswaran et al. [6] showed that black men younger than 39 years of age and those between 50–59 years of age had higher odds of an increased apnea hypopnea index compared to white men after controlling for body mass index. Similarly, Meetze et al. [7] compared four groups to assess if OSA severity differed by race/ethnicity. Among black men, adjusted analysis for body mass index and age, resulted in a higher mean respiratory disturbance index and lower mean oxygen saturation rate, suggesting that black men have more severe OSA compared to white men.

Absent from this body of evidence, however, is an assessment of the attitudes and beliefs toward sleep in this high-risk population. There is growing evidence that attitudes, perceptions, and beliefs about an individual’s illness are linked to health outcomes [8]. For instance, dysfunctions and rigidly-held beliefs about sleep are used to discriminate between good and poor sleepers [9].

Thus, it is also plausible that dysfunctional attitudes and maladaptive beliefs toward sleep could be related to risk perceptions of OSA and the optimal amount of sleep needed. Therefore, identifying attitudes and maladaptive beliefs about sleep could have important clinical implications for identifying black men at risk for OSA.

The present study recruited a sample of black men from barbershops in a large metropolitan area in Brookly, NY, to assess the maladaptive beliefs and attitudes about sleep. We hypothesized that maladaptive beliefs would be associated with men at high risk for OSA compared to men with low risk for OSA.

## Methods

Participants were a convenience sample of black adult men age 18 years or older (n=120) recruited from barbershops in Brooklyn, NY. Recruitment was conducted weekdays and weekends including evening hours. After introduction by the shop owner, research assistants introduced the study to customers on site to solicit participation. Once informed about the study, consenting participants completed self-administered questionnaires and provided sociodemographic and health data. Body mass index was based on self-reported height and weight, and was calculated as weight (kg)/height (m^2^).

To assess risk for OSA, we administered the Apnea Risk Evaluation System^™^ questionnaire (ARESTM), which includes questions on sleep patterns, daytime functioning, sleep apnea-related factors, and diseases associated with risk for sleep apnea (e.g., hypertension, diabetes mellitus).

ARESTM questionnaire data was used to identify men at high OSA risk (cut-off: >5). The questionnaire has a sensitivity of 0.94, specificity of 0.79 (based on a clinical cut-off of apnea-hypopnea index AHI>5), positive predictive value of 0.91, and negative predictive value of 0.86 [10]. Although there are other widely used tools including the STOP-Bang [11], we have used the ARESTM questionnaire to screen over 1,000 black patients in primary-care settings and 150 blacks in the community without documented refusals by participants, suggesting that it is feasible to administer this questionnaire in this population [12,13].

Participants completed the Dysfunctional Beliefs and Attitudes about Sleep scale-16 (DBAS-16) [14]. The scale assesses insomnia-related misconceptions. DBAS-16 is a Likert-type scale in which participants indicate a number ranging from 0 (strongly disagree) to 10 (strongly agree) in response to questions about beliefs and attitude toward sleep; the total score is an average of ratings on each of the 16 items.

A high score on the DBAS-16 indicates a high endorsement of dysfunctional beliefs and attitudes about sleep. The DBAS-16 has adequate internal consistency (Cronbach’s alpha 0.79) and temporal stability [14]. People who receive treatment for OSA who score high on the DBAS scale are more likely to have insomnia than people treated for OSA who score low on the DBAS scale [15].

Insomnia complaints were assessed by using the three commonly used questions in the field: “Do you have difficulty falling asleep?” (difficulty falling asleep); “Do you have difficulty staying asleep?” (difficulty maintaining sleep); and “Do you wake up earlier in the morning than you mean to?” (early morning awakening). Other sleep related measures included sleep duration, sleep medicine use, and naps during the day.

The study was approved by the Institutional Review Board at SUNY Downstate Medical Center.

## Analysis

We used frequency and measures of central tendency to describe the study sample. Contingency tables yielding Fisher’s exact tests were used to compare characteristics of participants with high and low OSA risk scores (Table 1). A one-way Analysis of Covariance (ANCOVA) was conducted to determine whether there were significant differences between insufficient (≤ 6 h) and healthy sleep (7–8 hours) in DBAS scores. In the ANCOVA model, we adjusted for the effects of age, body mass index, self-reported diagnosis of hypertension, diabetes mellitus, and mood and sleep variables. All statistical analyses were performed using SPSS version 18.

## Results

**Figure 1:**
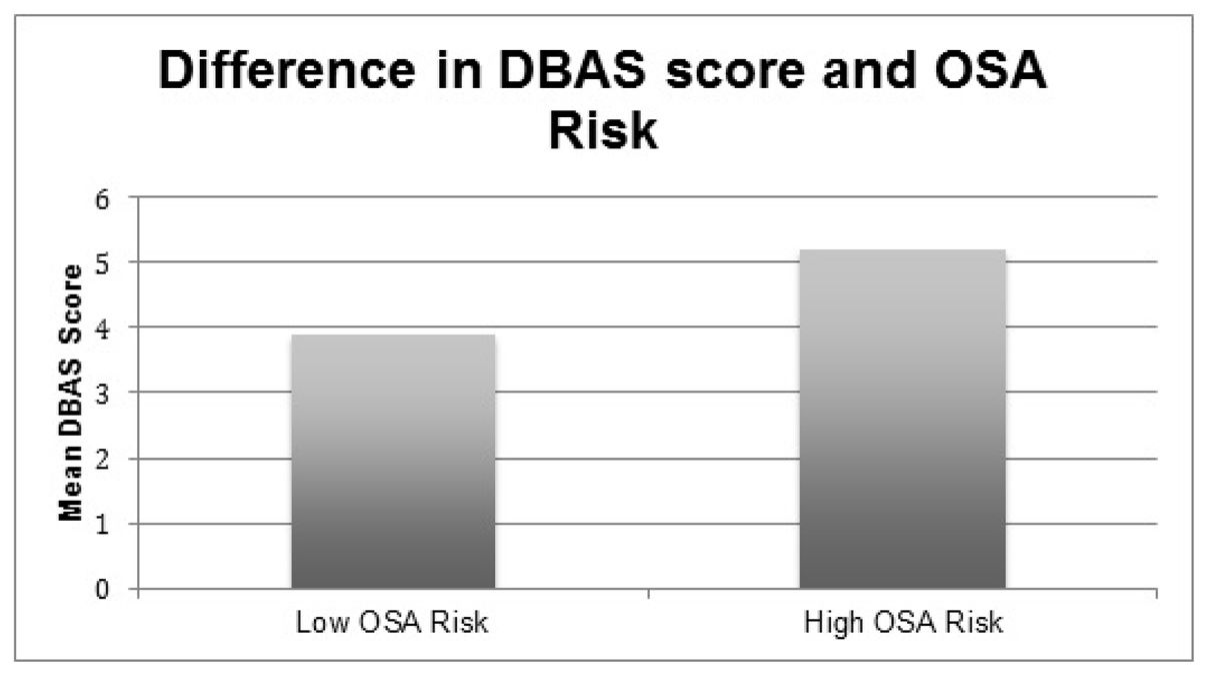
Difference in mean DBAS score between men with low OSA risk and high OSA risk adjusted for age, hypertension, heart disease, diabetes, body mass index, and hours of sleep.

The mean age of the sample was 42 ± 15 years. Participants experienced high unemployment and had a history of several chronic diseases. Specifically, 83% were unemployed, 68% were overweight or obese (BMI>25), 25% reported hypertension, 11% reported diabetes, and 3% reported heart disease. Regarding rates of self-reported sleep-related problems, 23% reported difficulty falling asleep, 23% reported difficulty maintaining sleep, 35% reported early morning awakening, and 6% reported the use of sleep medicine (Figure 1).

Whereas 57% reported insufficient sleep (≤ 6 h), 34% were satisfied with their sleep, 36% reported daytime napping, and 27% were at high OSA risk.

Table 1 provides sociodemographic and health data comparing men by OSA risk status. Compared with those at low OSA risk, black men at high risk of OSA were significantly more likely to be overweight/obese, to report a physician-rendered diagnosis of hypertension, to report taking naps, and to report any one of the insomnia symptoms The mean DBAS score was 4.27 ± 1.99.

Figure 1 illustrates the difference in the mean DBAS score, of men with low and high OSA risk adjusted for sociodemographic and health variables. In the ANCOVA results indicated that men at high OSA risk had greater DBAS scores [F1,129=7.73, p<0.001] and tended to report a greater rate of sleep dissatisfaction [46% vs. 13%, Χ2=24.52, p<0.001]. Participants who reported insufficient sleep did not have greater DBAS scores than healthy sleepers [F1,92=0.89, NS].

## Discussion

The main finding of our study is that participants at high risk for OSA endorsed maladaptive beliefs toward sleep and they also reported dissatisfaction with sleep. Previously reported results from focus groups with black men and women revealed sleep quality was not synonymous with or reflected in their overall health and wellbeing [16]. Therefore, it is plausible that maladaptive beliefs and lack of knowledge about the significance of sleep to overall health could contribute to poor symptom recognition. There is also evidence that blacks respond differently to the Epworth Sleepiness Scale, a commonly used measure in sleep clinics to assess symptoms of OSA [17]. Similarly, in a study among a sample of black patients exploring factors related to OSA assessment and referral, patients who communicated their sleep behaviors and practices with their physician were more likely to express a desire to undergo a sleep assessment [5].

A handful of studies have examined perceptions of OSA risk, but these studies largely focus on adherence to treatment for OSA [18–20]. None, to our knowledge, have focused on general perceptions about sleep. This is particularly important, as several studies have demonstrated that individuals who perceive that they are at risk of illness are more likely to engage in health-promoting behaviors [21]. Therefore, greater emphasis should be placed on cognitive factors that are likely to influence black men’s health-promoting behavior about healthy sleep practices.

Our findings are similar to another study that used DBAS and found that sleep-related cognitions occur across a wide-spectrum of sleep disorders including OSA and restless leg syndrome [22], In that study, patients diagnosed with sleep disorders other than insomnia scored high on the DBAS suggesting that dysfunctional beliefs about sleep present themselves in the context of sleep problems in general. This is an important finding as DBAS was developed used to discriminate between good and poor sleepers in a sample of insomniacs [23]. It remains a widely used tool in patients with insomnia and relatively underutilized with other sleep disorders. A few studies have used the DBAS in other patient populations, including patients with fibromyalgia [24] and patients with comorbid insomnia and mood disorders [25]. These studies also found high levels of maladaptive beliefs. In future studies, it would have been interesting to correlate self-reported symptoms with objective measures for OSA and insufficient sleep such as actigraphy measurements and maladaptive beliefs.

The second observation is that over half of participants in our study (51%) reported insufficient sleep, a consistent finding in the literature on sleep duration among blacks [26–28]. Moreover, 36% reported daytime napping and napping is associated with OSA [29]. Based on the ARESTM score, 27% were at high risk for OSA. Given that black men may experience symptoms of OSA at a younger age compared with White men [30], primary physicians could play an important role in communicating risk and encouraging objective screenings for OSA including referrals for home sleep testing and polysomnography.

The ARESTM was well accepted in this setting, but one must exercise caution as the US Preventive Task Force recently stated that there is insufficient evidence to recommend population level screening for OSA in asymptomatic patients [31]. In addition, the DBAS is not a clinical or screening tool, but may be very useful for understanding maladaptive beliefs toward sleep in the clinical setting.

The fact that sleep data in this study was self-reported, rather than measured with actigraph or other objective assessment could be considered as a limitation. However, self-reported data proved to be most acceptable to minority individuals in community-based samples that we studied [12,32]. Given that the study design was cross-sectional and used a convenience sample, causality could not be determined. It is also unclear whether these findings would be generalizable to other populations. In this study, confirming an OSA diagnosis with the use of home-based testing or polysomnography was not feasible. Despite these limitations, the study makes an important contribution to the sleep literature, as it provided data from black men, a population that is at high-risk for OSA, but is underrepresented in sleep health research in particular as well as the conduct of the research in a community-based setting. The setting for the study was successful in reaching black men and could be used effectively in future sleep health services research. Future studies should also consider further evaluation of sleep-related beliefs and practices among black men. It is plausible that black men may be more likely to endorse incorrect beleifs about sleep such as using alcohol for improved sleep quality or under-recognition of symptoms such as loud and habitual snoring.

The study sample was recruited exclusively from barbershops, and our results indicatat this is useful strategy for recruiting black men from a community-based setting. Additionally, it contributes further understanding regarding the relationships of OSA with both health status and sleep beliefs, which have not been previously examined.

## Figures and Tables

**Table 1:** Relationship between socio-demographic and health risk data based and OSA risk

Variable	OSA Low Risk	OSA High Risk
Body Mass Index		
Overweight/obese (%)	63	81*
Hypertension (%)	17	43*
Diabetes mellitus (%)	9	18
Heart disease (%)	1	9
Caffeine (%)	21	24
Alcohol (%)	27	32
Naps (%)	27	56*
Employed (%)	13	26
Difficulty falling asleep (%)	17	38*
Difficulty staying asleep (%)	39	24
Early wakening (%)	29	50*

* Indicates significant differences using Fisher’s Exact test at alpha=0.05
